# An improved suppression subtractive hybridization technique to develop species-specific repetitive sequences from *Erianthus arundinaceus* (*Saccharum* complex)

**DOI:** 10.1186/s12870-018-1471-6

**Published:** 2018-11-06

**Authors:** Fan Yu, Yongji Huang, Ling Luo, Xueting Li, Jiayun Wu, Rukai Chen, Muqing Zhang, Zuhu Deng

**Affiliations:** 1 National Engineering Research Center for Sugarcane, Fujian Agriculture and Forestry University, Fuzhou, 350002 Fujian China; 2 Guangdong Key Laboratory of Sugarcane Improvement and Biorefinery, Guangdong Provincial Bioengineering Institute, Guangzhou, China; 3 State Key Laboratory for protection and utilization of subtropical agro-bioresources, Guangxi University, Nanning, 530004 China; 4 Key Lab of Sugarcane Biology and Genetic Breeding, Ministry of Agriculture, Fujian Agriculture and Forestry University, Fuzhou, 350002 China

**Keywords:** Sugarcane, *E. arundinaceus*, Suppression subtractive hybridization, Fluorescence in situ hybridization, Species-specific repetitive sequences

## Abstract

**Background:**

Sugarcane has recently attracted increased attention for its potential as a source of bioethanol and methane. However, a narrow genetic base has limited germplasm enhancement of sugarcane. *Erianthus arundinaceus* is an important wild genetic resource that has many excellent traits for improving cultivated sugarcane via wide hybridization. Species-specific repetitive sequences are useful for identifying genome components and investigating chromosome inheritance in noblization between sugarcane and *E. arundinaceus*. Here, suppression subtractive hybridization (SSH) targeting *E. arundinaceus*-specific repetitive sequences was performed. The five critical components of the SSH reaction system, including enzyme digestion of genomic DNA (gDNA), adapters, digested gDNA concentrations, primer concentrations, and LA *Taq* polymerase concentrations, were improved using a stepwise optimization method to establish a SSH system suitable for obtaining *E. arundinaceus*-specific gDNA fragments.

**Results:**

Specificity of up to 85.42% was confirmed for the SSH method as measured by reverse dot blot (RDB) of an *E. arundinaceus* subtractive library. Furthermore, various repetitive sequences were obtained from the *E. arundinaceus* subtractive library via fluorescence in situ hybridization (FISH), including subtelomeric and centromeric regions. EaCEN2-166F/R and EaSUB1-127F/R primers were then designed as species-specific markers to accurately validate *E. arundinaceus* authenticity.

**Conclusions:**

This is the first report that *E. arundinaceus*-specific repetitive sequences were obtained via an improved SSH method*.* These results suggested that this novel SSH system could facilitate screening of species-specific repetitive sequences for species identification and provide a basis for development of similar applications for other plant species.

**Electronic supplementary material:**

The online version of this article (10.1186/s12870-018-1471-6) contains supplementary material, which is available to authorized users.

## Background

Rising energy demands are placing increasing pressure on finite oil reserves, with largely negative effects such as increased pollution and carbon emissions. Efforts have in expanded to increase the commercial production of ethanol, which is considered to be the most promising biofuel produced from renewable resources. The conversion of lignocellulosic materials into ethanol fuel is an attractive and promising method that involves renewable raw materials [1–3]. Among various agricultural crop byproducts, sugarcane bagasse is the most abundant lignocellulosic material in tropical countries and has been used for bioethanol and methane production [4, 5]. Sugarcane (*Saccharum* spp.) is mainly used for sugar production and as a clean energy substrate [6]. Unfortunately, the narrow genetic base of sugarcane has limited its resistance to face the possible impact of climate change on bioenergy production [7]. Hence, novel sugarcane varieties that have high yields and resistance to various stresses, both biotic and abiotic, are needed.

The genus *Saccharum* is an important member of the *Poaceae* family. The most widely considered concept by sugarcane breeder is that it consists of six species, including *Saccharum officinarum*, *Saccharum robustum*, *Saccharum spontaneum*, *Saccharum sinense*, *Saccharum barberi*, and *Saccharum edule* [8]. *S. spontaneum* is widely recognized as the most primitive species within the genus *Saccharum*, whereas *S. robustum* has been postulated to be the progenitor of the high sugar content species, *S. officinarum* [9]. These three species represent the basic species within the genus *Saccharum*. *S. sinense* and *S. barberi* are natural hybrids between *S. officinarum* and *S. spontaneum* [10, 11]. In addition, *S. edule* may be a cross between *Saccharum* and its closely related genus [12]. The genus *Saccharum* and its related wild genus, including *Miscanthus*, *Sclerostachya*, *Erianthus*, and *Narenga*, constitute the *Saccharum* complex [13], which is an important wild germplasm resource that can be used to broaden the genetic basis of sugarcane [14]. Several reports indicated that *Erianthus* belongs to *Saccharum* based on the presence/absence of awn whereas this is not exactly. The criteria to differentiate these two genera vary, but recent studies suggested that *Saccharum* and *Erianthus* are distinct genera based on various lines of molecular evidence [15–19]. As such, the *Saccharum* complex is broadly accepted to indicate the relationship between these species. *Erianthus arundinaceus* is a related wild species that has desirable agronomic traits for sugarcane improvement, such as high biomass, vigor, ratooning ability, drought tolerance, and water logging, as well as resistance to pests and disease [13, 20–23]. *E. arundinaceus* is already considered to be one of the most popular germplasm sources for crossing in sugarcane improvement. However, due to genomic divergence between sugarcane and closely related genera, the available molecular markers derived from closely related genera is limited. Therefore, species-specific repetitive sequences would be useful for identifying the genome or chromosome composition of interspecific or intergeneric hybrids and to monitor alien introgression in their progeny [24]. Interestingly, most species-specific markers have been identified as repetitive DNA sequences. Higher plants are known to contain large numbers of repetitive DNA sequences, which may be dispersed throughout the genome or tandemly arrayed at certain chromosome regions [25, 26]. Previously, repetitive DNA sequences were referred to as “junk DNA” because few discernible functions could be assigned to these regions [27]. However, an increasing amount of cytological and genomic sequencing data revealed that repetitive sequences play a significant role in chromosomal rearrangements, genomic differentiation and evolution [26]. Many of these repetitive DNA sequences have been identified as molecular markers in plants [28, 29]. For sugarcane, however, the amount of available sequence information that would allow the identification of molecular markers is limited and thus accurate identification at a species level that is fundamental for research on these plants is difficult.

A variety of approaches for plant species identification have been used, each varying in sensitivity, specificity, cost, and efficacy. Conventional methods of species identification mainly rely on morphology and physiological biochemistry, but the accuracy of these methods for species identification can be compromised by the influence of external environmental conditions. To overcome these challenges, genome-based approaches using polymerase chain reaction (PCR) and sequencing have shown promise as highly sensitive tools for species identification, including amplified fragmented length polymorphism (AFLP), arbitrarily primed PCR (AP-PCR), randomly amplified polymorphic DNA (RAPD), restriction length polymorphism (RFLP), inter simple sequence repeat (ISSR) anchored PCR, simple sequence repeat polymorphism (SSR), and single nucleotide polymorphism (SNP) [30–36]. However, timely and accurate species identification using these methods could not be made due to the long test period or inconsistent results [37]. In addition, genome sequencing can greatly facilitate the identification of species-specific repetitive sequences, although for sugarcane the cost and accuracy of genome sequencing is currently prohibitive due to its genomic complexity and that of related species [38]. Hence, a rapid and reliable identification method that allows both species identification and phenotype determination is needed to discover species-specific repetitive sequences.

Several molecular methods, including SSH, differential display PCR (DD-PCR) and representational difference analysis (RDA) have already been introduced into routine detection approaches or are currently under investigation for their performance in both species identification and phenotype characterization [39–41]. However, many organisms lack well-defined systems for determining lineage, and mapping remains difficult or impossible for genes that lack easily recognizable phenotypes. Among these methods, SSH, invented in 1996 by Diatchenko et al., is an effective method for isolation of specific DNA fragments that can be used to differentiate two closely related species [42–45]. A key feature of this method is simultaneous normalization and subtraction steps that respectively equalize the abundance of DNA fragments within the target population and exclude sequences common to the two populations being compared [43]. Species-unique gDNA fragments could be used as species-specific probes capable of distinguishing their ‘target’ species from all other species [46–50] and enable the profiling of genetic diversity in an environmental metagenome [51]. These advantages make SSH a rapid and accurate detection method that has been widely used for species identification. Li et al. [40] demonstrated that specific DNA fragments produced by SSH could be used as species-specific probes for the identification of five species of the genus *Dendrobium*. Ge et al. reported that genome-specific molecular markers obtained by SSH were applicable for detection of chromosomes or chromosomal fragments of *Lophopyrum elongatum* in a wheat background [52]. These studies provided valuable insights into gDNA subtraction between different species for obtaining tester-specific DNA sequences in plants using SSH. Considering the complex genetic background of modern sugarcane, which is both multiploid and aneuploid and has high number of chromosomes (~ 120), development of a rapid and effective method for species identification is critical.

The aim of this study was to establish a SSH technology optimization system for rapid and systematic screening of species-specific repetitive sequences of the entire genome from *E. arundinaceus*, a polyploid species. Furthermore, *E. arundinaceus*-specific repetitive sequences were used as a probe for physical mapping of *E. arundinaceus* chromosomes. According to chromosome location, two pairs of primers were designed to accurately identify *E. arundinaceus* authenticity. This improved SSH method is executable and efficient. It will be powerful for obtaining species-specific repetitive sequences in other plant for accurate and rapid identification of species. Additionally, these probes could be further used to establish *E. arundinaceus* chromosome karyotypes and investigate chromosome inheritance in the progeny of crosses between sugarcane and *E. arundinaceus* [53, 54]*.*

## Results

### The SSH optimization system Restriction enzyme digestion of *E. arundinaceus* gDNA

**Fig. 1:**
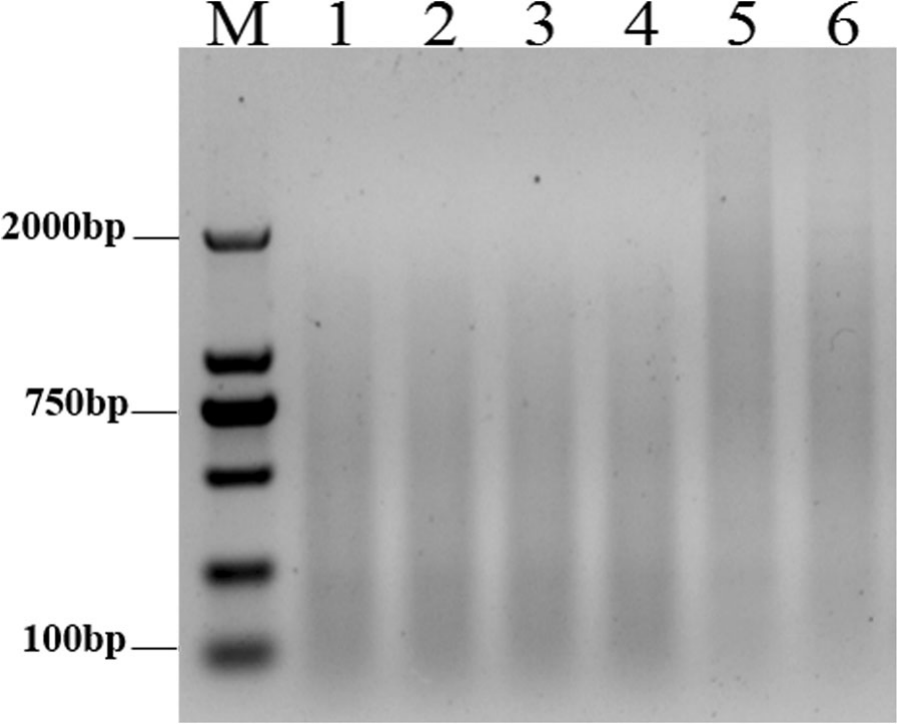
Enzyme digestion of genomic DNA. M: 2000 bp marker; *E. arundinaceus* gDNA double digested for 1 h (Lane 1); 2 h (Lane 2); 3 h (Lane 3); 4 h (Lane 4); *E. arundinaceus* gDNA digested for 4 h with *Hae*III (Lane 5) or *Alu*I (Lane 6)

To determine whether *E. arundinaceus* gDNA was completely digested, the digested gDNA was analyzed by agarose gel. Results showed that the size distribution of either *Hae*III or *Alu*I-digested gDNA was longer than 2 kb, even though the digestion time was as long as 4 h (Fig. 1, Lanes 5 and 6). Meanwhile, DNA double digested with both *Hae*III and *Alu*I showed a marked decrease in size to 0.1 to 2 kb, and appeared as a smear for all samples after 1 to 4 h of incubation (Fig. 1, Lanes 1 to 4), indicating that the digestion had progressed to completion after 1 h.

### Adapter ligation efficiency analysis

**Fig. 2:**
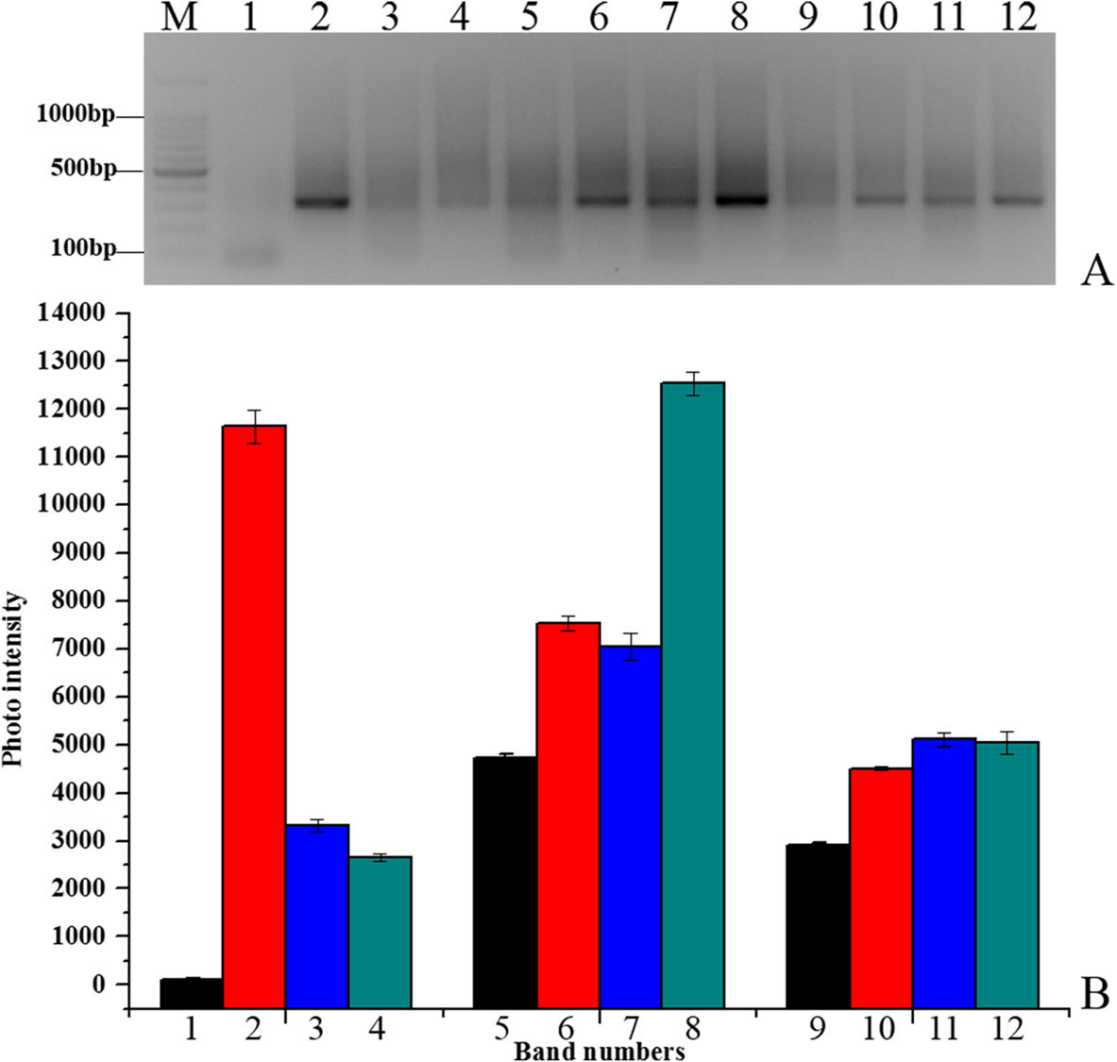
Adaptor ligation efficiency. Electrophoretic detection (**a**) and analysis of band intensity (**b**). M: 100 bp marker Lane 1: *Hae*III adapter 1 primer 1/204F; Lane 2: *Hae*III adapter 1 primer 1/204R; Lane 3: *Hae*III adapter 2R primer 1/204F; Lane 4: *Hae*III adapter 2R primer 1/204R; Lane 5: *Alu*I adapter 1 primer 1/204F; Lane 6: *Alu*I adapter 1 primer 1/204R; Lane 7: *Alu*I adapter 2R primer 1/204F; Lane 8: *Alu*I adapter 2R primer 1/204R; Lane 9: *RsaI* adapter 1 primer 1/204F; Lane 10: *RsaI* adapter 1 primer 1/204R; Lane 11: *RsaI* adapter 2R primer 1/204F; Lane 12: *RsaI* adapter 2R primer 1/204R

We designed three different adapters (*Rsa*I adapters, *Hae*III adapters, and *Alu*I adapters) to screen the appropriate adapter that could efficiently ligate with the digested gDNA. 28S-204F/R primers were designed to analyze the ligation efficiency of digested DNA. To avoid other factors that could influence ligation efficiency, four different primer combinations were used to the detect ligation efficiency of the different adapters. The *Alu*I adapters had the highest ligation efficiency relative to the *Hae*III adapters and *Rsa*I adapters (Fig. 2a). ImageJ analysis showed that the *Alu*I adapter bands had the highest average intensity as calculated from the average of signal intensities for three scans. Hence, *Alu*I adapters were used.

**Fig. 3:**
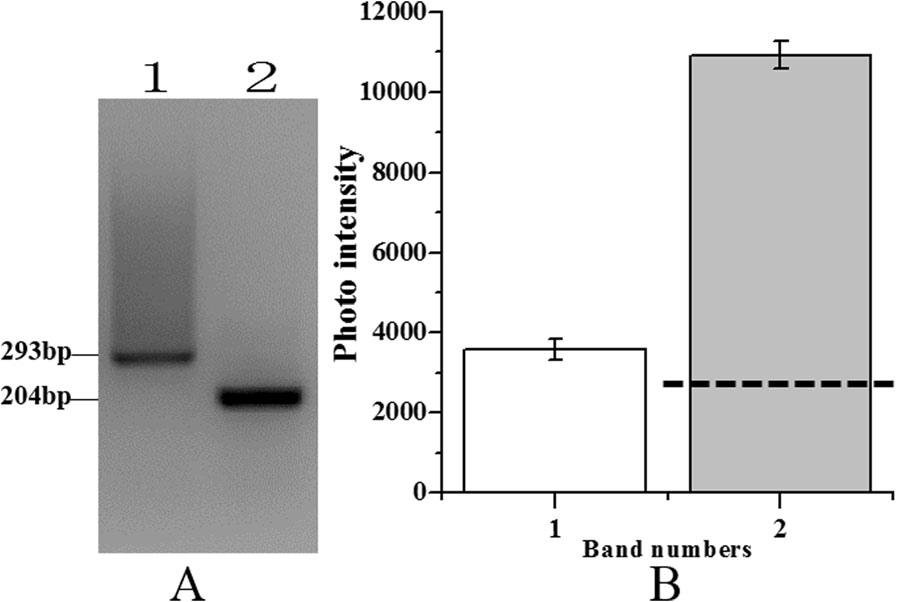
PCR analysis of adapter ligation efficiency analysis*.* Electrophoretic detection (**a**) and analysis of band intensity (**b**). M: 100 bp marker; Lane 1: 150 ng/μL experimental sample; Lane 2: 150 ng/μL control tester sample. Dashed line indicates 25% of control tester sample intensity

To determine the optimal concentration of the digested DNA to use for ligation reaction, four concentrations (50, 100, 150, and 200 ng) were used to ligate with the *Alu*I adapters. Among these different concentrations, the ligation efficiency was highest for 150 ng digested DNA (Additional file 1: Figure S1). Thus, 150 ng digested gDNA was used to ligate to the *Alu*I adapters and the reaction mix was incubated for 2 min at 72 °C in a thermal cycler to extend the adaptors. Gel electrophoresis showed that the experimental band that the *Alu*I adapters ligated with 28S rDNA fragment was 100 bp larger than the control tester DNA fragments without the *Alu*I adapters (Fig. 3a). ImageJ analysis indicated that the experimental band intensity was 25% greater than that of the control tester DNA fragments (Fig. 3b), suggesting at least 25% of the digested DNA fragments had adapters on both ends.

**Fig. 4:**
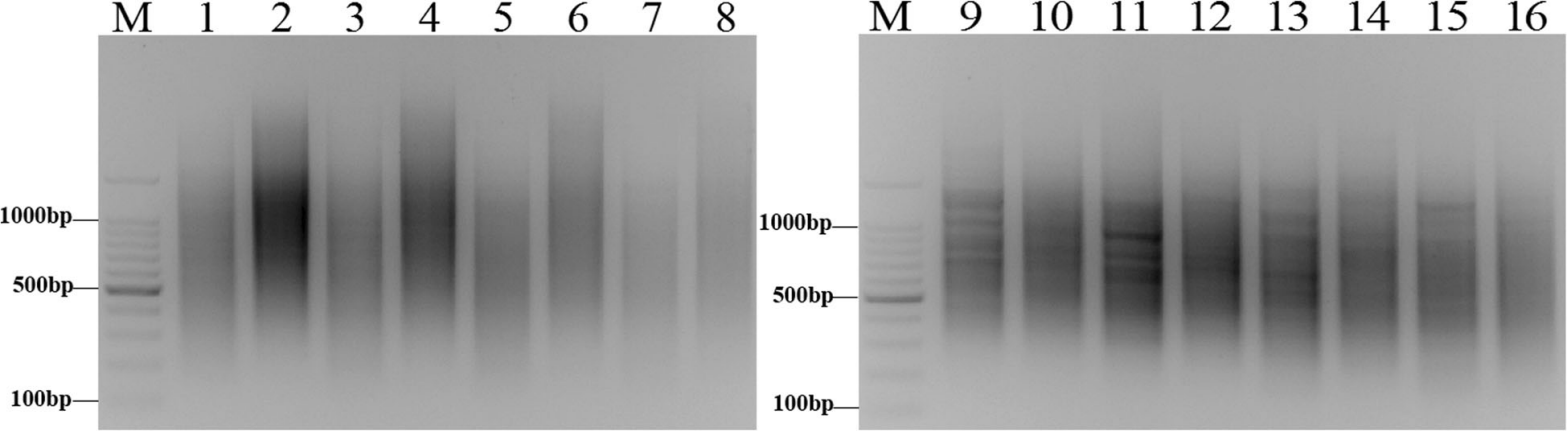
Electrophoresis of suppression PCR products after SSH. M: 100 bp marker; Lane 1: subtract first PCR 62 °C; Lane 2: unsubtract first PCR 62 °C; Lane 3: subtract first PCR 64 °C; Lane 4: unsubtracted first PCR 64 °C; Lane 5: subtracted first PCR 66 °C; Lane 6: unsubtracted first PCR 66 °C; Lane 7: subtracted first PCR 68 °C; Lane 8: unsubtracted first PCR 68 °C; Lane 9: subtracted second PCR 62 °C; Lane 10: unsubtracted second PCR 62 °C; Lane 11: subtracted second PCR 64 °C; Lane 12: unsubtracted second PCR 64 °C; Lane 13: subtracted second PCR 66 °C; Lane 14: unsubtracted second PCR 66 °C; Lane 15: subtracted second PCR 68 °C; Lane 16: unsubtracted second PCR 68 °C

To achieve optimal amplification efficiency, the two-step PCR reaction system was adjusted according to the primers used and LA *Taq* polymerase as described in the Materials and Methods. Primary PCR and secondary PCR were performed with different temperature gradients ranging from 62 °C to 68 °C. In primary PCR, two primer concentrations (10 and 20 μM) and two LA *Taq* polymerase concentrations (0.5 and 1 U) were used for PCR amplification (Additional file 2: Figure S2a-c and Fig. 4). Among the concentrations tested, 20 μM primer with 1 U LA *Taq* polymerase yielded the best results (Fig. 4). The unsubtracted products appeared as a diffuse band of 0.2–2 kb, whereas the subtracted products appeared as a smear that had weak intensity and a smaller size (Fig. 4). In secondary PCR, two primer concentrations (20 and 30 μM) and two LA *Taq* polymerase concentrations (0.5 and 1 U) were used for PCR amplification (Additional file 3: Figure S3a-c and Fig. 4), with 30 μM primer and 1 U LA *Taq* polymerase providing complete amplification (Fig. 4). Both the unsubtractive and subtractive products appeared as a diffuse band, whereas the subtracted products appeared as a number of distinct bands (Fig. 4). This result demonstrated that subtractive products were successfully obtained using the improved amplification system.

### The efficiency of SSH PCR products

**Fig. 5:**
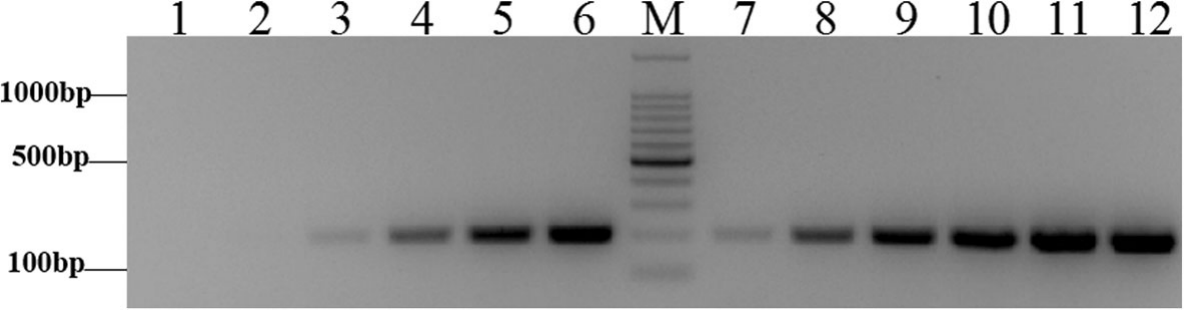
SSH efficiency. M: 100 bp marker; Lanes 1–6: products of subtracted DNA by 18, 21, 24, 27, 30, and 33 cycles; Lanes 7–12: products of unsubtracted DNA by 18, 21, 24, 27, 30, and 33 cycles

Subtractive efficiency performed by PCR detection using 28S-204F/R primers showed that the unsubtracted product appeared as a weak band after 18 cycles, whereas the subtracted product did not produce an obvious band until 24 cycles were reached (Fig. 5). This result showed that 28S rRNA gene reduction was successfully achieved in a control sugarcane gDNA sample (Fig. 5).

As the cloning and screening efficiency using this method was unknown, up to 192 (two 96-well plates) white color clones were selected from an *E. arundinaceus* subtractive library. To eliminate as many false positive clones as possible, the nest primer set 1/2R rather than the M13F/R primer set was used to amplify the selected clones, so that the recombinant clones were amplified by PCR. A total of 135 positive clones were obtained from a subtractive library of *E. arundinaceus*. The ratio of positive clones was 70.31%, and the clones were 60–1000 bp. This result verified that the improved SSH system was suitable for cloning into the pMD19-T vector and that the clones were incorporated with high efficiency.

### Screening *E. arundinaceus*-specific sequences by RDB

**Fig. 6:**
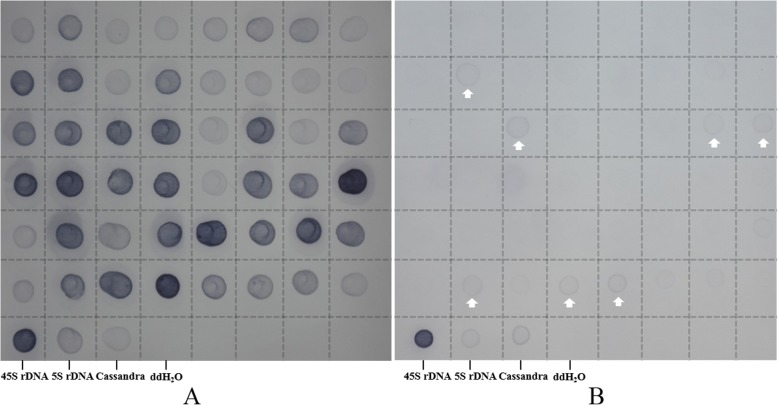
RDB hybridization of *E. arundinaceus*-specific sequences. 48 probes were printed in gDNA of *E. arundinaceus* (**a**) and sugarcane (**b**). Arrows indicate non-specific positive

To obtain proof of principle for SSH specificity, RDB was performed by hybridization with *E. arundinaceus* (Fig. 6a) and sugarcane (Fig. 6b) gDNA. As predicted, obvious signals for the 45S rDNA, 5S rDNA and Cassandra positive controls were observed, whereas little or no signal was observed for the negative control using ddH_2_O. Thus, the hybridizations were successful. In an *E. arundinaceus* subtractive library, 41 *E. arundinaceus*-specific clones were obtained from 48 clones that represented different sizes among the 135 positive clones, and signals were only observed for *E. arundinaceus* (Fig. 6a). The RDB results showed that the improved SSH technology yielded an *E. arundinaceus*-specific rate of up to 85.42% in a subtractive library between *E. arundinaceus* and sugarcane.

### Localization of *E. arundinaceus*-specific repetitive sequences in *E. arundinaceus* chromosomes using FISH

**Fig. 7:**
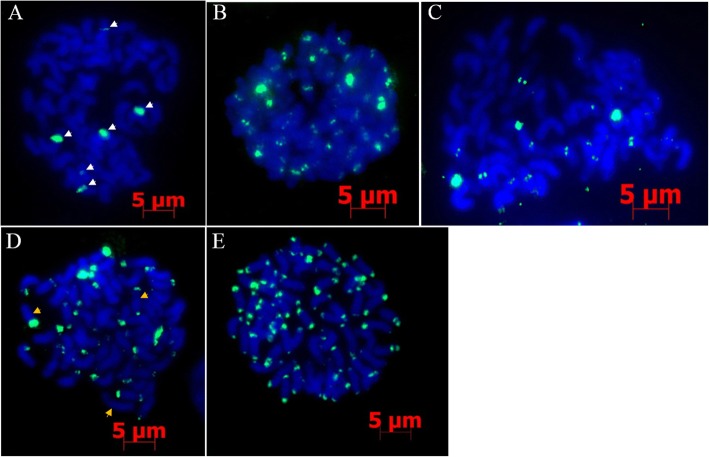
FISH mapping of *E. arundinaceus*-specific probes in Hainan 92–77. Five *E. arundinaceus*-specific probes, EaCEN1 (**a**), EaCEN2 (**b**), EaCEN3 (**c**), EaSUB2 (**d**), EaSUB1 (**e**), were mapped to metaphase chromosomes. Specific FISH signals were detected using these five repeat probes. White arrows indicate six signals and three signal types, respectively. Bar = 5 μm

FISH was performed using chromosome preparations made from Hainan 92–77 root tips and 41 *E. arundinaceus*-specific DNA sequences as probes. FISH analysis to locate *E. arundinaceus* chromosomes showed two location types, in centromeric regions (Fig. 7a-c, Additional file 4: Table S1) and subtelomeric regions (Fig. 7d, e, Additional file 4: Table S1). Interestingly, EaCEN1 only had six signal sites on the chromosomes (Fig. 7a), whereas EaSUB2 localized to the subtelomeric regions at one or both ends of most chromosomes. Some chromosomes showed no hybridization signal (Fig. 7d). This result indicated that various repetitive sequences could be obtained from the *E. arundinaceus* subtractive library that could be used for karyotype analysis of *E. arundinaceus*.

### Species identification with species-specific primers

**Fig. 8:**
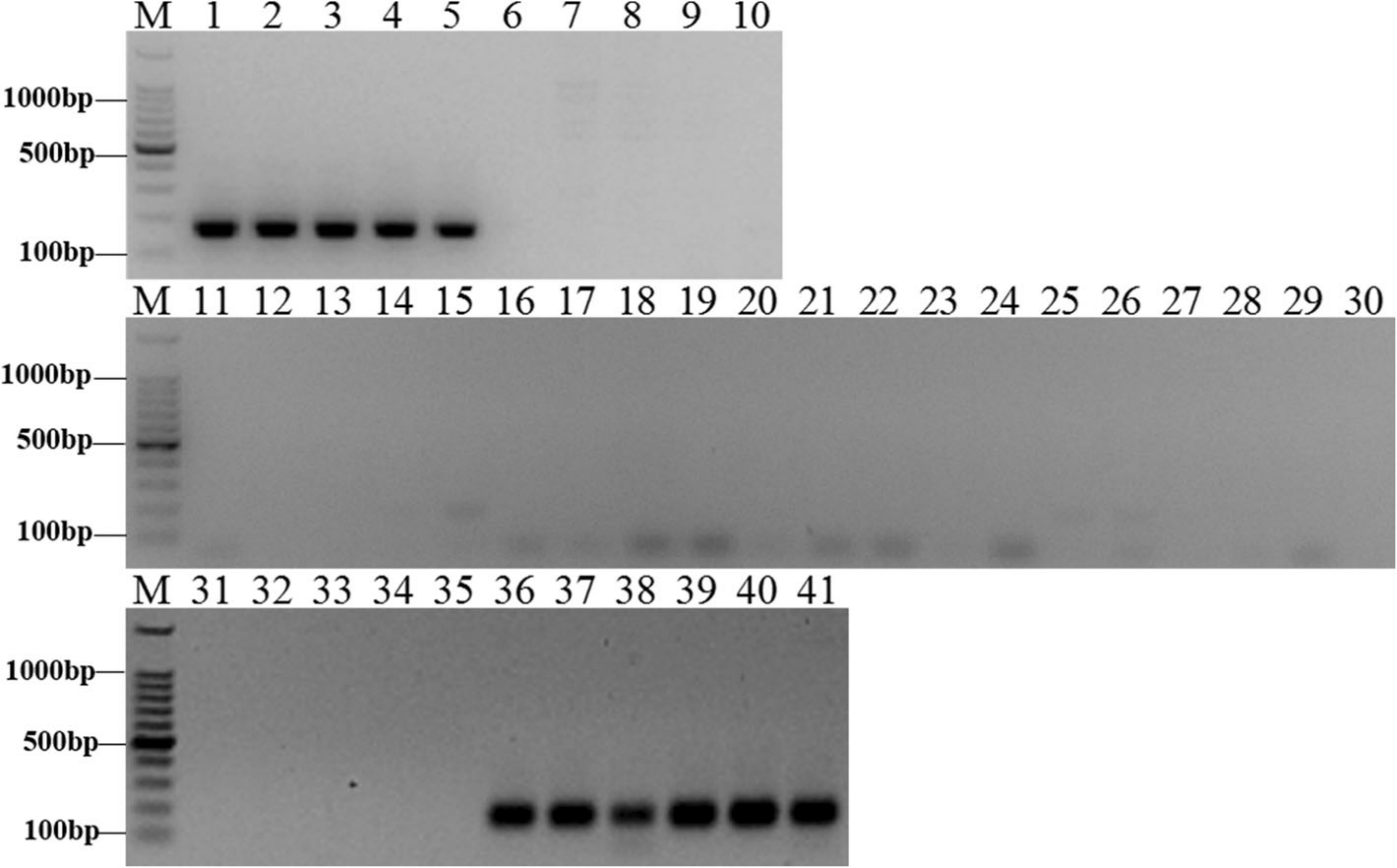
Amplification profile of the specific primer EaCEN2-166F/R. M: 100 bp marker; Lane 1: Hainan 92–77; Lane 2: Hainan 92–105; Lane 3: Yunnan 83–180; Lane 4: Yunnan 82–30; Lane 5: Yunnan 82–80; Lane 6: Badila; Lane 7: Loethers; Lane 8: Luohan Zhe; Lane 9: Vietnam Niuzhe; Lane 10: Black Cheribon; Lane 11: 51NG3; Lane 12: NG77–004; Lane 13: 51NG63; Lane 14: 57NG208; Lane 15: Daye; Lane 16: Yunnan 75–2-11; Lane 17: Fujian 89–1-19; Lane 18: Fujian 92–1-11; Lane 19: Fujian 89–1-18; Lane 20: Yunnan 82–50; Lane 21: Hetang Zhuzhe; Lane 22: Wenshan Zhuzhe; Lane 23: Uba; Lane 24: Guangdong Zhuzhe; Lane 25: Guangxi Zhuzhe; Lane 26: Nagans; Lane 27: Panshi; Lane 28: Katha; Lane 29: Saretha; Lane 30: Mungo; Lane 31: R570; Lane 32: ROC22; Lane 33: CP84–1198; Lane 34: ROC16; Lane 35: ROC10; Lane 36:Yacheng96–40; Lane 37:Yacheng96–41; Lane 38:Yacheng96–43; Lane 39:Yacheng96–45; Lane 40:Yacheng96–66; Lane 41:Yacheng96–69

**Fig. 9:**
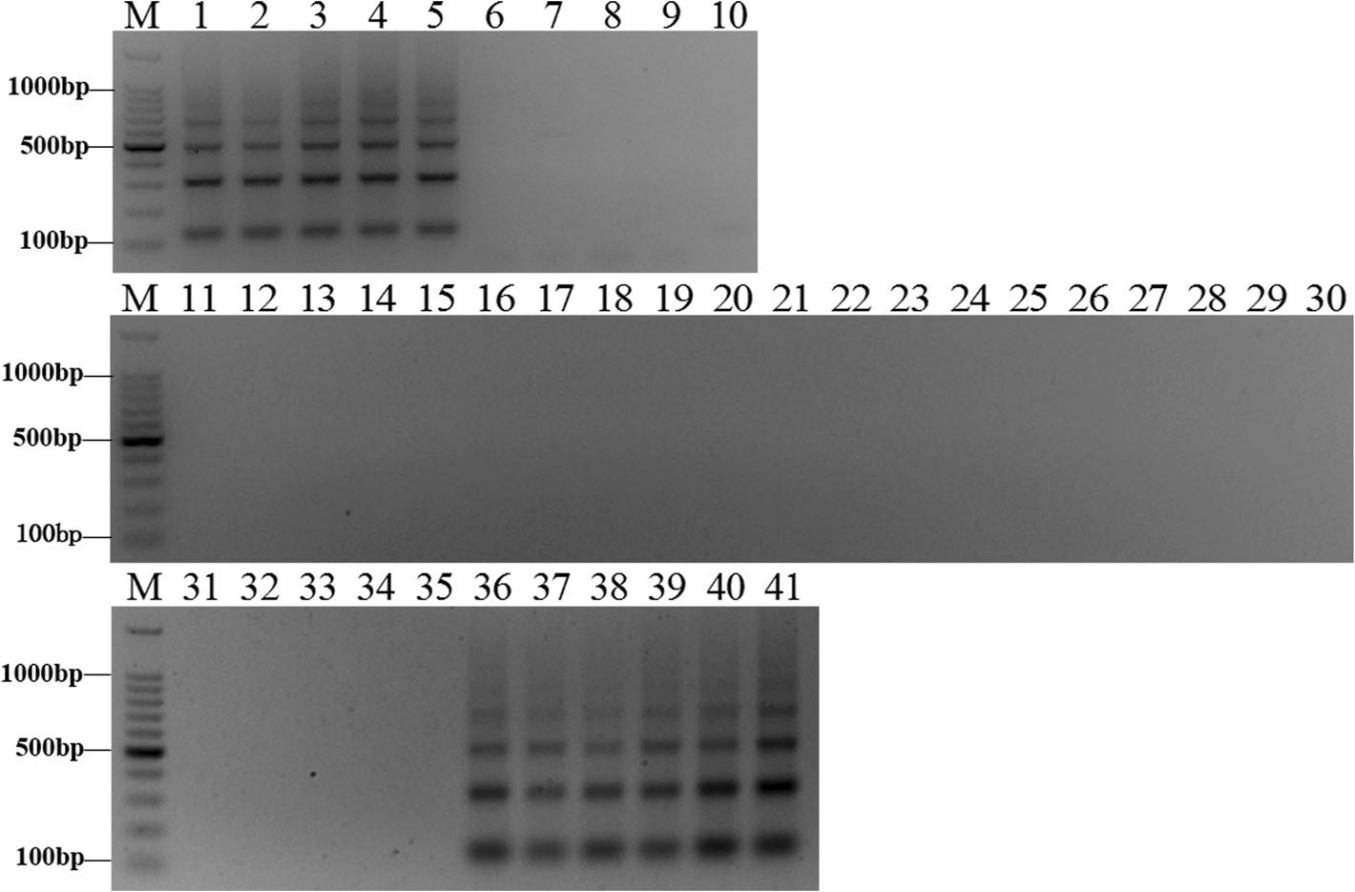
Amplification profile of the specific primer EaSUB1-127F/R. M: 100 bp marker; Lane 1: Hainan 92–77; Lane 2: Hainan 92–105; Lane 3: Yunnan 83–180; Lane 4: Yunnan 82–30; Lane 5: Yunnan 82–80; Lane 6: Badila; Lane 7: Loethers; Lane 8: Luohan Zhe; Lane 9: Vietnam Niuzhe; Lane 10: Black Cheribon; Lane 11: 51NG3; Lane 12: NG77–004; Lane 13: 51NG63; Lane 14: 57NG208; Lane 15: Daye; Lane 16: Yunnan 75–2-11; Lane 17: Fujian 89–1-19; Lane 18: Fujian 92–1-11; Lane 19: Fujian 89–1-18; Lane 20: Yunnan 82–50; Lane 21: Hetang Zhuzhe; Lane 22: Wenshan Zhuzhe; Lane 23: Uba; Lane 24: Guangdong Zhuzhe; Lane 25: Guangxi Zhuzhe; Lane 26: Nagans; Lane 27: Panshi; Lane 28: Katha; Lane 29: Saretha; Lane 30: Mungo; Lane 31: R570; Lane 32: ROC22; Lane 33: CP84–1198; Lane 34: ROC16; Lane 35: ROC10; Lane 36:Yacheng96–40; Lane 37:Yacheng96–41; Lane 38:Yacheng96–43; Lane 39:Yacheng96–45; Lane 40:Yacheng96–66; Lane 41:Yacheng96–69

Combined with the FISH results, two sequences were selected to design primers to target the subtelomeric and centromeric regions. To confirm the validity of the species-specific primers, species identification experiments were carried out by PCR using *E. arundinaceus*-specific primers to amplify *E. arundinaceus* and sugarcane gDNA. Three primer pairs were designed according to EaCEN2 and EaSUB1, and one pair of primers was obtained (Additional file 4: Table S1). PCR amplification with EaCEN2-166F/R showed that the expected bands were only observed in five *E. arundinaceus* samples and six *E. arundinaceus*-derived F_1_ hybrids samples (Fig. 8), and a similar result was obtained using EaSUB1-127F/R (Fig. 9). Thus, these primers could be used to identify authentic *E. arundinaceus* and sugarcane hybrids.

## Discussion

### Advantages of SSH technology to screen species-specific DNA fragments and identify species

*E. arundinaceus* has significant potential as a germplasm source for sugarcane breeding. There are multiple reports of successful cross-hybridization between *E. arundinaceus* and sugarcane [53, 54] and the various resulting progeny showed broad chromosome translocations between *Saccharum* spp. and *E. arundinaceus* [55]. Thus, a routine and reliable approach to identify *E. arundinaceus*-derived hybrids is needed to improve the efficiency of sugarcane breeding. SSH is a widely used technology to obtain species-specific sequences and this approach forms the foundation for many other methods [56–60]. Furthermore, a significant number of studies and applications have demonstrated the feasibility of SSH in plants [61, 62]. Currently, SSH has been used to select species-specific sequences in only a few plants. However, SSH affords many unique advantages, including high sensitivity, high specificity, low false positive rate and abundant differential sequences [40, 52]. In this study, we developed an improved SSH system to produce *E. arundinaceus*-specific repetitive sequences between sugarcane and *E. arundinaceus*. Findings from this study indicated that this improved SSH method is an inexpensive and efficient tool to capture *E. arundinaceus*-specific repetitive sequences, and thus this approach could have substantial potential for further identification of species-specific repetitive sequences in other plants. Since higher plants contain many repetitive sequences, during PCR amplification high copy number and short sequences are first amplified, whereas sequences having a low copy number and long sequences have limited amplification. Thus, sequences having a high copy number and shorter lengths are more easily obtained in a SSH library due to this migration enrichment [63]. The phenomenon of high copy number of migration is in accord with the aim of this study, which was to enrich repetitive sequences in *E. arundinaceus*. Our method not only reduced the frequency of low copy number sequences, but also enriched moderately and highly repetitive sequences. Although the final library contained a few copies of DNA fragments, these can be screened out by RDB. Therefore, many species-specific repetitive sequences could be obtained. We chose two *E. arundinaceus*-specific repetitive sequences located in subtelomeric and centromeric regions to design two primers, EaCEN2-166F/R and EaSUB1-127F/R, respectively. Each primer pair accurately identified the authenticity of *E. arundinaceus* among 41 samples.

### Key factors for obtaining specific DNA sequences with SSH

Originally, SSH was used to screen differentially expressed genes in cDNA and has since been applied to screen differential DNA fragments in gDNA. Unlike cDNA, gDNA is larger and has a more complex structure [64]. Digestion of complete gDNA with restriction enzymes can be used to reduce this complexity and obtain appropriately sized fragments. At the same copy number, longer DNA fragments have higher C_o_t-values compared to shorter fragments [65]. Hence, longer DNA fragments require an increased renaturation time, and insufficient hybridization could produce false positives. In contrast, shorter DNA fragments can be amplified with preference in PCR. Therefore, we digested gDNA with two restriction enzymes to ensure that the digested fragments were around 2 kb. This approach could also effectively eliminate secondary structures present in long DNA fragments. In this study, the digested fragments ranged between 0.1 and 2.0 kb, which effectively improved the likelihood of differentiating *E. arundinaceus* and sugarcane DNA sequences. Additionally, the connection efficiency of adapters played an important role in obtaining different gDNA fragments. An intensity lower than 25% of the control connection efficiency could result in loss of gDNA fragments. Therefore, instead of *Rsa*I gDNA digestion, we used both *Hae*III and *Alu*I. The adapter sequences were then modified to ensure ligation efficiency, with *Alu*I*-*adapters found to have the highest efficiency.

Thorough hybridization subtraction was also critical for improving the specific positive rate and doubling of the hybridization time produced different outcomes. In earlier studies, the first hybridization time typically ranged from 6 to 12 h, whereas the second was between 12 and 24 h [45, 63]. In this study, the two hybridization times were 8 h and 17 h, respectively, thus almost completely avoiding recombination of single DNA fragments in the first hybridization and ensuring that low amounts of single-stranded DNA were able to form double-stranded DNA in the second hybridization. In addition, the proportion of DNA in the drive group and test group was at least 20:1 to remove homologous DNA sequences in the two distantly related species [63]. Here, we used this ratio for hybridization that allowed the subtraction of homologous DNA sequences between *E. arundinaceus* and sugarcane. If this approach is used to separate closely related species, the ratio of the driver group to the test group should be increased.

### Development and utilization of *E. arundinaceus*-specific repetitive sequence probes in FISH

FISH technology can directly display the position of DNA sequences on chromosomes with high sensitivity and accuracy, which has increased the importance of this technique for studies of molecular and cellular genetics in plants [66]. Repetitive sequence probes can be combined with FISH to track chromosomal inheritance and analyze karyotypes. To date, C_o_t-1 DNA, Bacterial Artificial Chromosome (BAC) library, Yeast Artificial Chromosome (YAC) library and chromatin immunoprecipitation (ChIP) methods have been widely applied in combination with FISH to study plant genomes [67–73]. These methods provide tools to obtain chromosome markers that are directly based on their genomic sequences. However, none have been used as a specific chromosome marker method for species-specific chromosomal tracking, possibly due to the use of non-specific probes. In our study, we obtained several specific repetitive sequences using the SSH method. These sequences could be used to track chromosomes from *E. arundinaceus-*derived hybrids. In particular, six signal sites were detected in Hainan 92–77 using the EaCEN1 probe. D’Hont et al. used pTa71 probes in *E. arundinaceus* with 2n = 60 chromosomes to show that the basic chromosome number was x = 10 [20]. This finding indicates that these chromosomes were homologous to chromosomes in *E. arundinaceus* and can be used to analyze specific-chromosome inheritance in offspring resulting from crosses between *E. arundinaceus* and sugarcane. Additionally, repetitive sequences have been used as a probe to identify chromosomes in plants. Indeed, Kato et al. filtered tandem repeat DNA sequences from a random PCR library to generate a distinctive banding pattern for each of the 10 chromosomes in maize [74]. Meanwhile, Zhang et al. also used repetitive sequence probes to enable unequivocal identification of each of the 21 homologous chromosomes in wheat [32]. Here, several *E. arundinaceus*-specific repetitive sequences were obtained from the *E. arundinaceus* subtractive library. These probes were positioned in subtelomeric or centromeric regions, and the signal numbers varied. This outcome could lay the foundation for identifying chromosome karyotypes for *E. arundinaceus* in the future.

## Conclusions

The improved SSH method presented here is shown to be a highly effective and reliable approach for obtaining *E. arundinaceus*-specific repetitive sequences between sugarcane and *E. arundinaceus* that have large and complex genome. Compared with other methods to detect species-specific repetitive sequences that can be used for identifying species lineages, this method has many advantages in that it is simple, rapid, accurate and inexpensive. A key feature of this method is that it can simultaneously capture larger numbers of species-specific sequences from the whole genome. The size and complexity of nucleic acids in genomes of higher eukaryotes produces a reservoir of abundant DNA molecular markers. Our results demonstrated that the ratio of *E. arundinaceus*-specific sequences from screened subtracted clones reached 85.42%, indicating that the improved SSH allows simple screening of larger species-specific probes for definite species authentication. Notably, many tester-specific fragments were *E. arundinaceus*-specific repetitive sequences. Various species-specific repetitive sequences are helpful for exact species identification and karyotype analysis. This method has the potential to improve the efficiency of authentication studies between sugarcane and *E. arundinaceus*. Moreover, the repetitive fragments of *E. arundinaceus* -specific sequences will aid chromosome studies for *E. arundinaceus*.

## Methods

### Plant material and gDNA preparation

Six different species, sugarcane cultivars and F_1_ hybrids between *E. arundinaceus* and *S. officinarum* provided by the Research Institute Ruili Station, the Sugarcane Research Institute of Yunnan Agriculture Science Academy, as well as the Hainan Sugarcane Breeding Station, Guangzhou Sugarcane Industry Research Institute were used in this study (Table 1). Leaf tissues from these materials were ground in liquid nitrogen and stored at − 80 °C. Total genomic DNA (gDNA) was extracted from young leaves following an improved cetyltrimethyl ammonium bromide (CTAB) methodology [75].

**Table 1:** Experimental materials

No.	Accession	Species	No.	Accession	Species
1	Hainan 92–77	*E. arundinaceus*	22	Wenshan Zhuzhe	*S. sinense*
2	Hainan 92–105	*E. arundinaceus*	23	Uba	*S. sinense*
3	Yunnan 82–30	*E. arundinaceus*	24	Guangdong Zhuzhe	*S. sinense*
4	Yunnan 82–80	*E. arundinaceus*	25	Guangxi Zhuzhe	*S. sinense*
5	Yunnan 83–180	*E. arundinaceus*	26	Nagans	*S. barberi*
6	Badila	*S. officinarum*	27	Panshi	*S. barberi*
7	Yuenan Niuzhe	*S. officinarum*	28	Katha	*S. barberi*
8	Black Cheribon	*S. officinarum*	29	Saretha	*S. barberi*
9	Loethers	*S. officinarum*	30	Mungo	*S. barberi*
10	Luohan Zhe	*S. officinarum*	31	R570	*S. officinarum*, *S. spontaneum, S. barberi*
11	51NG3	*S. robustum*	32	ROC22	*S. officinarum*, *S. spontaneum, S. barberi*, *S. robustum*
12	NG77–004	*S. robustum*	33	CP84–1198	*S. officinarum*, *S. spontaneum, S. barberi*
13	51NG63	*S. robustum*	34	ROC16	*S. officinarum*, *S. spontaneum, S. barberi*, *S. robustum*
14	57NG208	*S. robustum*	35	ROC10	*S. officinarum*, *S. spontaneum*, *S. barberi*, *S. robustum*
15	Daye	*S. robustum*	36	Yacheng96–40	*S. officinarum*, *E. arundinaceus*
16	Yunnan 75–2-11	*S. spontaneum*	37	Yacheng96–41	*S. officinarum*, *E. arundinaceus*
17	Fujian 89–1-19	*S. spontaneum*	38	Yacheng96–43	*S. officinarum*, *E. arundinaceus*
18	Fujian 92–1-11	*S. spontaneum*	39	Yacheng96–45	*S. officinarum*, *E. arundinaceus*
19	Fujian 89–1-18	*S. spontaneum*	40	Yacheng96–66	*S. officinarum*, *E. arundinaceus*
20	Yunnan 82–50	*S. spontaneum*	41	Yacheng96–69	*S. officinarum*, *E. arundinaceus*
21	Hetang Zhuzhe	*S. sinense*			

### SSH and differential DNA fragment cloning

SSH was used to isolate DNA fragments present in the target plant materials but absent from the reference clones. The procedure was performed using the PCR-Select Bacterial Genome Subtraction Kit (Clontech), with modifications for *E. arundinaceus* and sugarcane. Three basic species of the genus *Saccharum*, *S. officinarum*, *S. robustum* and *S. spontaneum* (gDNA pooled in 1:1:1 ratio) were assigned as the SSH driver whereas *E. arundinaceus* was assigned as the SSH tester. Tester and driver samples were digested with *Hae*III, *Alu*I, or both. Adapter ligations were performed using 100 ng digested gDNA that was ligated to 40 μM *Rsa*I-adapter1/2R, *Hae*III-adapter1/2R, and *Alu*-adapter1/2R by 700 U T4 DNA ligation in 10 μL reactions. Ligation efficiency was analyzed using 28S-204F/R primers, which were designed according to the conservative 28S rDNA sequence of plants. Then, for obtaining the optimal ligation efficiency, four digested gDNA concentrations (50, 100, 150, and 200 ng) were ligated to *Alu*-adapter1/2R. PCR amplification of tester-specific fragments was performed using primers directed to tester ligated adapter sequences. A Veriti® 96-Well Thermal Cycler (ABI, USA) was used for both primary and secondary PCR amplifications. Based on the initial conditions described in the PCR-Select Bacterial Genome Subtraction Kit instruction, primary PCR amplifications were performed using two primer1 (5’-CTAATACGACTCACTATAGGGC-3′) concentrations (10 and 20 μM) and two LA *Taq* polymerase concentrations (0.5 and 1 U) in a 25 μL reaction that included 2 μL hybridization products, 2.5 μL 10 × LA buffer, 2 μL 2.5 mM dNTP mixture (TaKaRa LA Taq™, Takara Biotechnology, Inc.). Secondary PCR amplifications were performed using nest primer 1: 5’-TCGAGCGGCCGCCCGGGCAGAG-3′ and nest primer 2R: 5’-AGCGTGGTCGCGGCCGAGAG-3′ in a 25 μL reaction volume that included 1 μL of 10-fold diluted primary PCR products, 2.5 μL 10 × LA buffer, 2 μL dNTP mixture (2.5 mM), two primer concentrations (20 and 30 μM), and two LA *Taq* polymerase concentrations (0.5 and 1 U). The primary PCR cycling conditions were: one cycle at 72 °C for 8 min to fill in adapters and incubation at 94 °C for 5 min followed by 30 cycles of 94 °C for 30 s, 66 °C for 30 s and 72 °C for 90 s, and ending with a 5 min extension and storage at 4 °C. Secondary PCR cycling involved a similar program, but used an annealing temperature of 68 °C. Secondary PCR products were purified using a QIAquick PCR purification kit (Qiagen Inc.) and ligation into the pMD19-T-vector (Qiagen, Inc.). Plasmid DNA was purified using a Plasmid Mini kit I (200) (OMEGA) and then quantified using a NanoVue Plus (GE Healthcare, UK). DNA sequencing was performed by Beijing Genomics Institute (BGI) Co., Ltd. (Shenzhen, China).

### Preparation of DIG-labeled probes and RDB

*E. arundinaceus* and sugarcane gDNA was labeled by nick translation with digoxigenin (DIG) (Roche, Switzerland). Reverse dot blots (RDB) were performed to detect *E. arundinaceus*-specific clones. Purified plasmids (50 ng/μL) were denatured by heating to 97 °C for 10 min and quickly chilled in an ice/water bath for 15 min. The denatured plasmids were transferred onto Amersham Hybond-NC nylon membranes (GE Healthcare, Life Sciences, Indianapolis, IN, United States). Each plasmid (1 μL) was spotted onto the membranes, and DNA was fixed to the membrane by UV crosslink using a Stratalinker™ UV Crosslinker (Stratagene, La Jolla, CA, United States). After fixation, the membrane was pre-hybridized for 30 min. RDBs were carried out using a DIG High Prime DNA Labeling and Detection Starter Kit I (Roche, USA) according to the manufacturer’s instructions, with slight modifications. High stringency washes were performed following a rinse in wash solution containing 0.2 × saline-sodium citrate (SSC) and 0.1% sodium dodecyl sulfate before the blots were washed twice at 68 °C for 15 min each. Hybridization signals were detected with ChemiDocXRS (Bio-Rad, Hercules, CA, United States).

### FISH and primer sequences

Root tips were obtained from Hainan 92–77. Chromosomal preparations and FISH were performed as described by D’Hont et al. [76]. DIG-labeled differential DNA probes were prepared by PCR reaction with nest primer 1/2R in a Veriti® 96-Well Thermal Cycler (ABI, USA). Reactions were performed using a PCR-DIG Probe Synthesis Kit (Roche Diagnostics) according to the manufacturer’s instructions, under the following cycling conditions: denaturation for 5 min at 94 °C; 35 cycles of 30 s at 94 °C, 30 s at 68 °C, 90 s at 72 °C; and a final extension for 5 min at 72 °C. DIG-labeled differential DNA fragments were used as candidate probes for screening species-unique repetitive sequence probes on metaphase chromosomes from Hainan 92–77. Chromosomes were counterstained with 4′, 6-diamidino-2-phenylindole (DAPI) in a Vectashield anti-fade solution (Vector Laboratories, Burlingame, CA). Detection of DIG with fluorescein isothiocyanate (FITC) and amplification were performed as described by D’Hont et al. [76]. FISH signals were captured using the AxioVision measurement module of an Axio Scope A1 Imager fluorescent microscope (Zeiss, Germany). Based on the FISH results, two primers were designed to accurately identify *E. arundinaceus* authenticity (Additional file 4: Table S1).

## Additional files

Additional file 1: **Figure S1.** Ligation efficiency of different concentrations of digested gDNA. All lanes include *Alu*I adapter 1 primer 1/204F, *Alu*I adapter 1 primer 1/204R, *Alu*I adapter 2R primer 1/204F, and *Alu*I adapter 2R primer 1/204R with the indicated amount of digested gDNA. Lanes 1–4: 50 ng digested gDNA; Lanes 5–8: 100 ng digested gDNA; Lanes 9–12: 150 ng digested gDNA; Lanes 13–16: 200 ng digested gDNA. (TIF 10089 kb)

Additional file 2: **Figure S2.** Electrophoresis of primary PCR products after SSH. M: 100 bp marker; Lanes 1–4: products of subtracted first PCR by 62 °C, 64 °C, 66 °C, 68 °C. 10 μM primers and 0.5 U LA *Taq* polymerase (a), 10 μM primers and 1 U LA *Taq* polymerase (b), 20 μM primers and 0.5 U LA *Taq* polymerase (c). (TIF 12107 kb)

Additional file 3: **Figure S3.** Electrophoresis of secondary PCR products after SSH. M: 100 bp marker; Lanes 1–4: products of subtracted second PCR by 62 °C, 64 °C, 66 °C, 68 °C. 20 μM primers and 0.5 U LA *Taq* polymerase (a), 20 μM primers and 1 U LA *Taq* polymerase (b), 30 μM primers and 0.5 U LA *Taq* polymerase (c). (TIF 11035 kb)

Additional file 4: **Table S1.** Nucleotide sequences used in this study. (XLSX 9 kb)

## Abbreviations

CTAB: cetyltrimethyl ammonium bromide
DAPI: 4′, 6-diamidino-2-phenylindole
DIG: digoxigenin
FISH: fluorescence in situ hybridization
FITC: fluorescein isothiocyanate; SSC: saline-sodium citrate
gDNA: genomic DNA
PCR: polymerase chain reaction
RDB: reverse dot blot
SSH: suppression subtractive hybridization
