# Supplementary figures and images for: An improved suppression subtractive hybridization technique to develop species-specific repetitive sequences from Erianthus arundinaceus (Saccharum complex)
Source: BMC Plant Biol. 2018 Nov 6;18:269. doi: 10.1186/s12870-018-1471-6 (PMC6220460; doi:10.1186/s12870-018-1471-6)

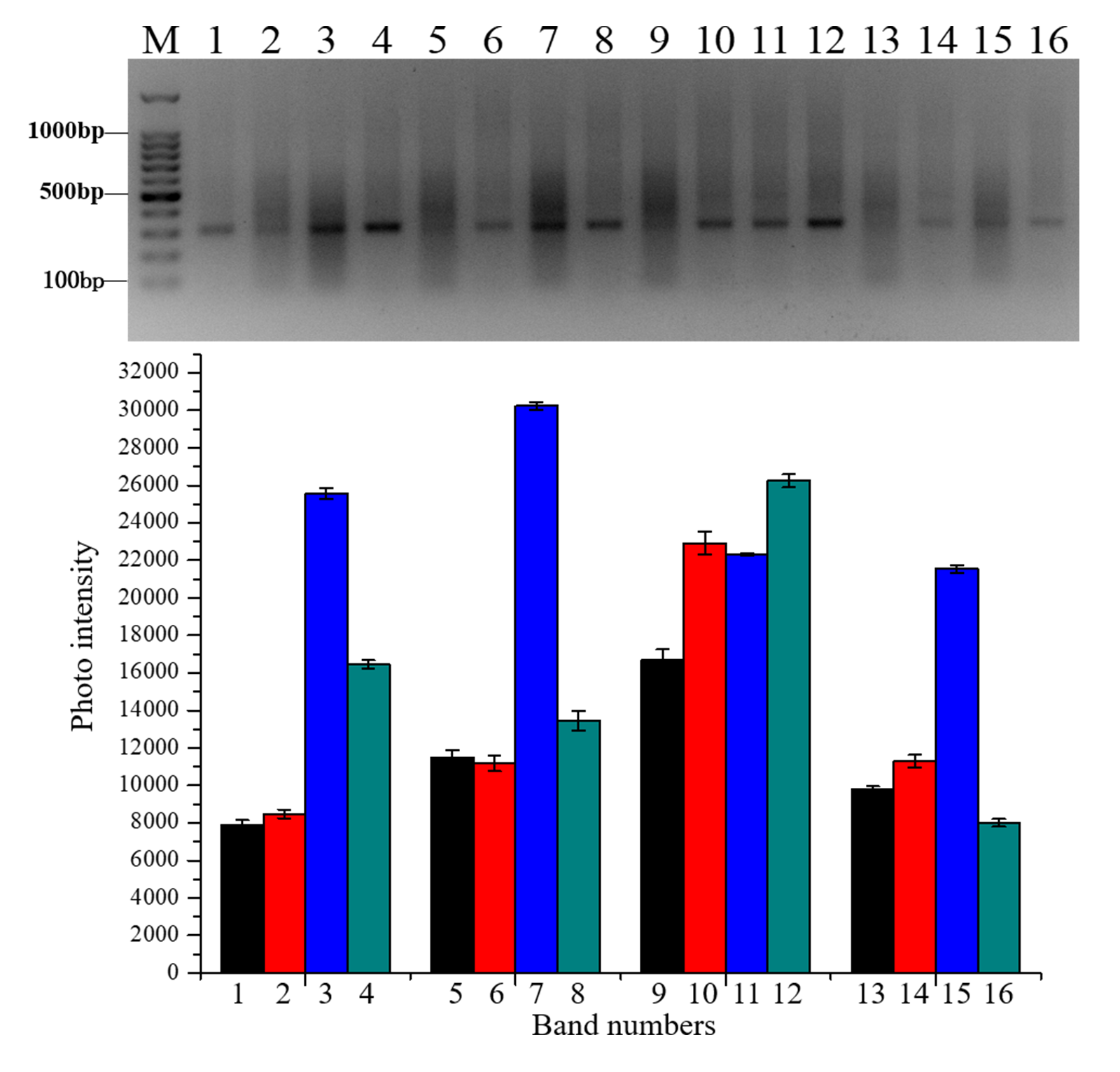

Supplement: Supplementary file 1 — Figure S1. Ligation efficiency of different concentrations of digested gDNA. All lanes include AluI adapter 1 primer 1/204F, AluI adapter 1 primer 1/204R, AluI adapter 2R primer 1/204F, and AluI adapter 2R primer 1/204R with the indicated amount of digested gDNA. Lanes 1–4: 50 ng digested gDNA; Lanes 5–8: 100 ng digested gDNA; Lanes 9–12: 150 ng digested gDNA; Lanes 13–16: 200 ng digested gDNA. (TIF 10089 kb) [file 12870_2018_1471_MOESM1_ESM.tif]

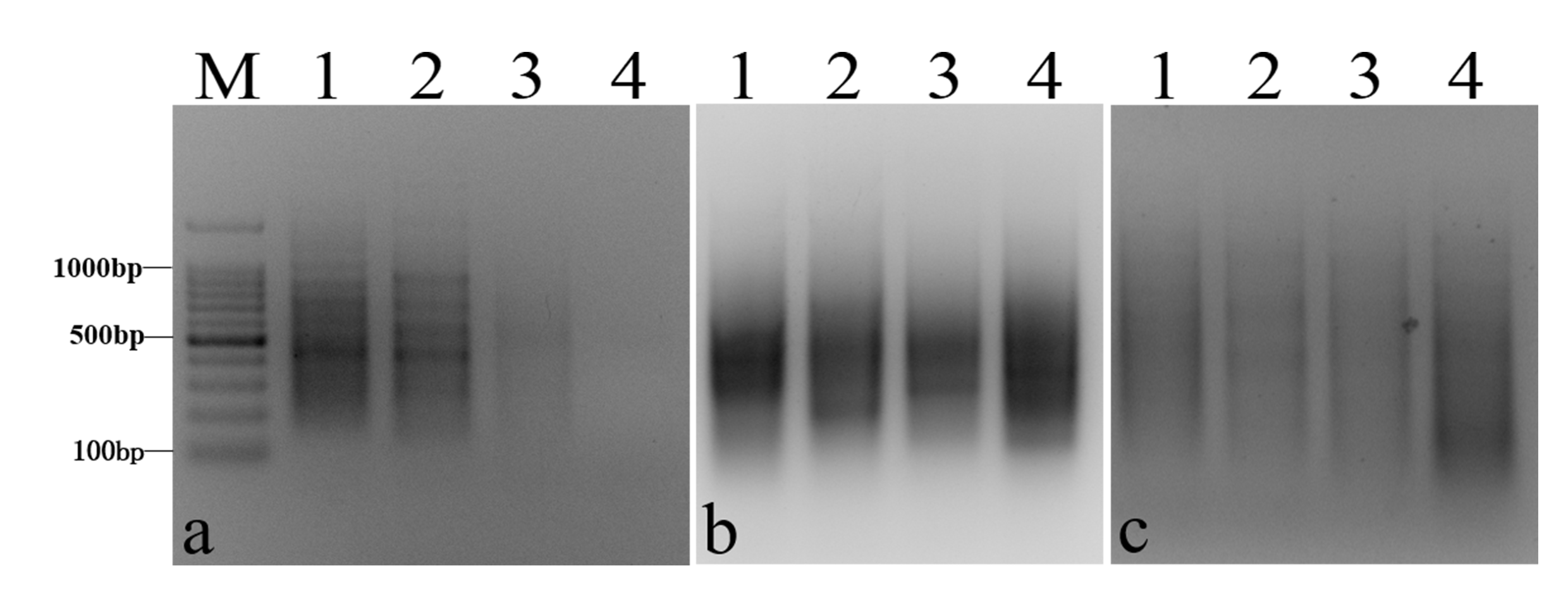

Supplement: Supplementary file 2 — Figure S2. Electrophoresis of primary PCR products after SSH. M: 100 bp marker; Lanes 1–4: products of subtracted first PCR by 62 °C, 64 °C, 66 °C, 68 °C. 10 μM primers and 0.5 U LA Taq polymerase (a), 10 μM primers and 1 U LA Taq polymerase (b), 20 μM primers and 0.5 U LA Taq polymerase (c). (TIF 12107 kb) [file 12870_2018_1471_MOESM2_ESM.tif]

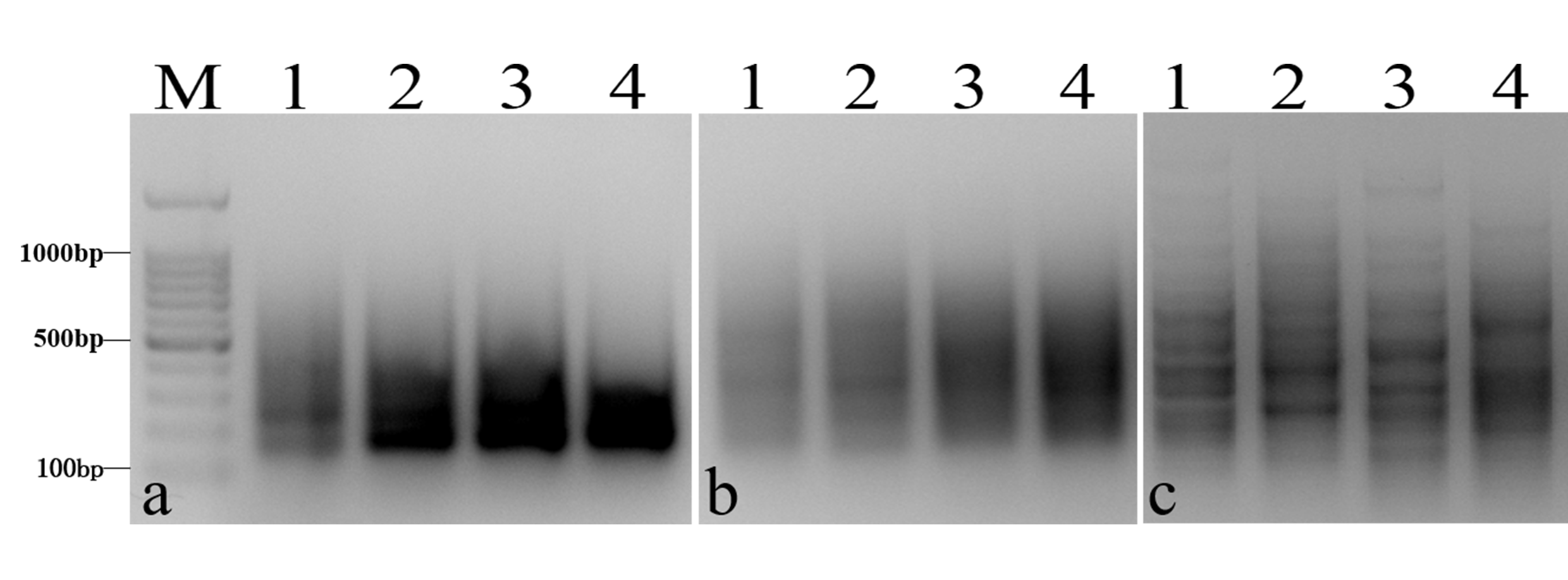

Supplement: Supplementary file 3 — Figure S3. Electrophoresis of secondary PCR products after SSH. M: 100 bp marker; Lanes 1–4: products of subtracted second PCR by 62 °C, 64 °C, 66 °C, 68 °C. 20 μM primers and 0.5 U LA Taq polymerase (a), 20 μM primers and 1 U LA Taq polymerase (b), 30 μM primers and 0.5 U LA Taq polymerase (c). (TIF 11035 kb) [file 12870_2018_1471_MOESM3_ESM.tif]
